# Phenolic Compounds Exerting Lipid-Regulatory, Anti-Inflammatory and Epigenetic Effects as Complementary Treatments in Cardiovascular Diseases

**DOI:** 10.3390/biom10040641

**Published:** 2020-04-21

**Authors:** Laura Toma, Gabriela Maria Sanda, Loredan Stefan Niculescu, Mariana Deleanu, Anca Volumnia Sima, Camelia Sorina Stancu

**Affiliations:** 1 Lipidomics Department, Institute of Cellular Biology and Pathology “Nicolae Simionescu” of the Romanian Academy, 8, B.P. Hasdeu Street, 050568 Bucharest, Romania; laura.toma@icbp.ro (L.T.); gabriela.sanda@icbp.ro (G.M.S.); loredan.niculescu@icbp.ro (L.S.N.); mariana.deleanu@icbp.ro (M.D.); anca.sima@icbp.ro (A.V.S.); 2 Faculty of Biotechnology, University of Agronomical Sciences and Veterinary Medicine, 59, Marasti Blvd., 011464 Bucharest, Romania

**Keywords:** cardiovascular diseases, inflammation, lipid metabolism, non-coding RNA, oxidative stress, phenolic compounds

## Abstract

Atherosclerosis is the main process behind cardiovascular diseases (CVD), maladies which continue to be responsible for up to 70% of death worldwide. Despite the ongoing development of new and potent drugs, their incomplete efficacy, partial intolerance and numerous side effects make the search for new alternatives worthwhile. The focus of the scientific world turned to the potential of natural active compounds to prevent and treat CVD. Essential for effective prevention or treatment based on phytochemicals is to know their mechanisms of action according to their bioavailability and dosage. The present review is focused on the latest data about phenolic compounds and aims to collect and correlate the reliable existing knowledge concerning their molecular mechanisms of action to counteract important risk factors that contribute to the initiation and development of atherosclerosis: dyslipidemia, and oxidative and inflammatory-stress. The selection of phenolic compounds was made to prove their multiple benefic effects and endorse them as CVD remedies, complementary to allopathic drugs. The review also highlights some aspects that still need clear scientific explanations and draws up some new molecular approaches to validate phenolic compounds for CVD complementary therapy in the near future.

## 1. Introduction

Cardiovascular diseases (CVD) continue to be the leading cause of mortality and morbidity worldwide, despite the various therapies developed for their treatment, therapies that have an important global economic impact [1,2]. The main cause of CVD is atherosclerosis, a multifactorial disorder induced and augmented by risk factors such as dyslipidemia, oxidative and inflammatory stress, diabetes mellitus, hypertension, smoking, ageing and genetic mutations [3]. Many therapies designed to treat atherosclerosis either have failed completely or partially (cholesteryl ester transfer protein (CETP) inhibitors, antioxidants, vitamins), or were too expensive to be applied to the entire population at CVD risk (apoA-I Milano); others, though successful, induced considerable side-effects (statins) [4,5]. Thus, the quest for strategies to prevent or treat CVD is of high and continued interest. In the last decade the scientific researchers turned their attention to phytochemicals, as effective, safe and low-cost natural bioactive compounds for CVD treatment.

Dyslipidemia consists of increased blood concentrations of total cholesterol (TC), low density lipoproteins–cholesterol (LDL-C) and/or triglycerides (TG), and decreased high density lipoproteins–cholesterol (HDL-C) [6]. The lipid metabolism is complex and the candidate mechanisms that could generate dyslipidemia include: (i) excessive dietary lipid absorption in the small intestine; (ii) packing of exogenous lipids with cholesterol and fatty acids produced de novo in the liver and their secretion as very low density lipoproteins (VLDL); (iii) hydrolysis of TG from VLDL by lipases and their conversion into LDL, which are taken up by the peripheral tissues through LDL receptor (LDL-R) and scavenger receptors; (iv) diminished production of HDL by the liver and small intestine, thereby decreasing reverse cholesterol transport (RCT) from the peripheral tissues to the liver; (v) lowered excess cholesterol excretion from the liver into gallbladder or to the intestinal lumen through the ATP-binding cassette G5 and G8 transporters (ABCG5/G8) that facilitate trans-intestinal cholesterol efflux (TICE). Dyslipidemia is associated with the accumulation of LDL in the sub-endothelium of the artery wall. At this site, LDL undergoes oxidative modifications (oxLDL) that trigger inflammatory responses, and is taken up by the monocyte-derived macrophages infiltrated in the sub-endothelium which thus become lipid-loaded foam cells, the hallmark of atheroma development [7]. Until now, the most effective lipid-lowering treatment for hyperlipidemic patients was the statin therapy. But recent recommendations have extended it to the asymptomatic adults at low cardiovascular risk (Systematic COronary Risk Evaluation <1%) and with lower LDL-C levels (<4.9 mmol/L), according to their levels of predicted CVD risk [8]. This extension will be accompanied by a higher number of subjects manifesting side effects and the need for replacement of the statin therapy with new effective and better tolerated medication.

Under physiological conditions, reactive oxygen species (ROS) are generated in low concentrations and serve as mediators that regulate vascular function [9,10]. Increased generation of ROS, due to the increased production of free radicals or paucity of antioxidants, generates oxidative stress and causes peroxidation of cellular lipids and lipoproteins that contribute to CVD progression [11]. Oxidative stress affects the bioavailability of nitric oxide (NO), an important vascular relaxing factor. When the endothelial nitric oxide synthase (eNOS) is uncoupled, NO and the superoxide radicals react and generate the oxidizing peroxynitrite anions, thereby inducing endothelial dysfunction [12]. Mitochondria control cell energy production and the respiratory cycle, processes closely related to oxidative stress [13]. To counteract or prevent the detrimental effect of ROS, numerous antioxidant systems have been developed, such as glutathione, flavonoids, superoxide dismutase (SOD), catalase, glutathione peroxidase (GSH-Px) and hemeoxygenase-1 (HO-1) [14]. The nuclear erythroid 2-related transcription factor (Nrf2) is redox-sensitive and plays an important role in regulating the production of the antioxidant enzymes [15]. Under physiological conditions, the enzyme paraoxonase 1 (PON1) accounts for HDL antioxidant properties, while the pro-oxidant enzyme myeloperoxidase (MPO) becomes attached to HDL under hyperlipidemic conditions [16]. In the last few decades, the quality and function of HDL became a consistent indicator of CVD risk [17,18]. Under augmented oxidative stress, HDL becomes dysfunctional, presenting an increased number of MPO molecules that replace the PON1 molecules [17,19]. Thus, dysfunctional HDL can no longer protect LDL from oxidation, these alterations being the key risk factors for initiation and progression of the atherosclerotic plaques.

Exposure of endothelial cells (EC) to risk factors induces their activation which consists in the production of pro-inflammatory molecules such as selectins (E-selectin, P-selectin, L-selectin), and of adhesion molecules, such as intracellular adhesion molecule 1 (ICAM-1) and vascular cell adhesion 1 (VCAM-1) promoting monocyte adhesion and their transmigration into the sub-endothelium [20,21]. The macrophages produce pro-inflammatory cytokines, such as interleukin-1β (IL-1β), IL-12 and tumor necrosis factor-α (TNF-α), or chemokines, such as monocyte chemoattractant protein-1 (MCP-1) to attract more monocytes, thereby stimulating the inflammatory process [22]. A central role in the stimulation of the inflammatory stress in atherosclerosis is played by the nuclear factor kappa B (NF-κB) [23]. A strong inhibitor of NF-κB is sirtuin-1 (SIRT-1), and toll-like receptor 4 (TLR4) is an activator of NF-κB [21,24]. Another important pro-inflammatory intracellular protein complex is the nucleotide binding and domain-like receptor 3 (NLRP3) inflammasome, which stimulates the primary pro-inflammatory response determining the activation of the secondary inflammatory mediators, such as IL-6 or C reactive protein (CRP), thereby amplifying the inflammatory stress [25].

Since the completion of the Human Genome Project, it has become evident that several additional mechanisms related to epigenomic, transcriptomic, epitranscriptomic, proteomic and metabolomic regulations are crucial for determinations of the phenotypes of many human disorders, including CVD [26]. The emerging non-coding RNAs (ncRNAs) have major regulatory roles in gene expression, and thus could function as potential targets for personalized treatment of CVD patients [27,28,29]. They can be divided into small ncRNAs (<200 nt), such as microRNA (miRNAs), transfer RNAs and small nucleolar RNAs, and longer ncRNAs (lncRNA), that include ribosomal and natural antisense transcripts. Members of a class of small (~22 nt) ncRNA, single stranded in mature form miRNAs have been identified as potent post-transcriptional regulators of genes, including those involved in lipid metabolism [30]. MiRNAs control their target gene’s expression by either imperfect base pairing to 3′ untranslated regions (3′UTR) of mRNA [31] or by binding to other regions, including 5′ UTRs or protein-coding exons [32], thereby inducing the repression of their target mRNAs. Circulating miRNAs are found in the blood closely associated with proteins, lipoproteins and extracellular vesicles (EVs) [33]. The levels of circulating miRNAs vary and have specific profiles in diverse pathophysiological states [34,35], leading to the possibility of using these molecules as promising markers for diagnosis and prognosis [36,37]. Published studies demonstrate that plasma miRNAs may be used as markers in the diagnosis of myocardial infarction [38], while EVs and HDL-associated miRNAs levels can discriminate between stable and vulnerable coronary artery disease patients [39,40,41]. It was reported that circulating and hepatic miRNAs expression could be modulated by hypolipidemic dietary interventions, such as probiotics administration [42]. For all these reasons, miRNAs could be very useful targets in nutritional science and could be used to test the pathways modulated by dietary treatments in healthy and/or diseased populations [43].

Phytochemicals such as phenolic compounds have been extensively studied, and important data exist on their use in CVD patients. In the present review we aim to focus on the molecular mechanisms of action that prove the multiple benefic effects of the phenolic compounds and endorse them as CVD remedies, complementary to allopathic drugs. We explore the active compounds that effectively target dyslipidemia, and oxidative and inflammatory stress, the main risk factors in atherosclerosis evolution. Thus, we discuss below the representative compounds for which a consistent number of mechanisms of action have been described. They belong to the following groups: hydroxycinnamic acids (curcumin, caffeic acid), stilbenes (resveratrol), flavonols (quercetin), flavones (apigenin, luteolin), flavanones (naringenin, hesperetin), flavanols (catechins, gallocatechins), isoflavones (genistein), anthocyanins/anthocyanidines and guaiacols (gingerols, shogaols).

## 2. Biologically Active Phenolic Compounds and Their Mechanisms of Action

Phytochemicals are biologically active compounds from plants that can regulate physiological and pathological processes with benefic consequences for human health. Phenolic compounds are one of the largest groups of phytochemicals. Their bioavailability is decisive to exerting beneficial effects in vivo and is influenced by the molecular size and complexity of their chemical structure, including conjugation with other phenols, polymerization, glycosylation, acylation or hydroxylation [44].

### 2.1. Hydroxycinnamic Acids Group

**Figure 1:**
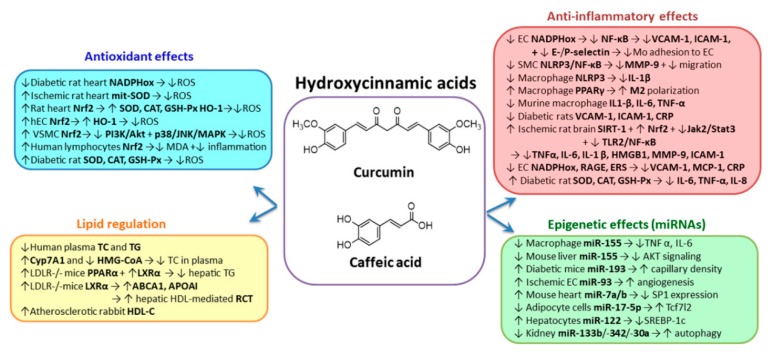
Chemical structure and protective effects exerted by curcumin and caffeic acid to improve cardiovascular diseases outcomes as demonstrated by experimental and clinical evidence.

*Curcumin* (diferuloylmethane) (Figure 1) is the most important bioactive compound from *Curcuma longa*, belonging to the *Zingiberaceae* family. The curcuminoids comprise several compounds, such as curcumin, desmethoxycurcumin and bis-demethoxy-curcumin. The source of curcumin is turmeric, a yellow-colored spice [45]. Pharmacokinetic studies revealed that curcumin is poorly soluble in water; has low absorption in the gut, rapid metabolism and systemic elimination, and consequently, has low bioavailability after oral administration. The clinical efficacy of curcumin could be improved by formulations that enhance its solubility and stability and diminish the first-pass metabolism. To that end, certain strategies have been elaborated, such as the development of curcumin–piperine complexes, curcumin nanoparticles, cyclodextrin inclusions, curcumin liposomes and curcumin phospholipids’ complexes, part of these systems exhibiting a 100-fold increase of bioavailability relative to unformulated curcumin [46].

In vitro and in vivo studies demonstrate curcumin’s pleiotropic effects, due to its ability to interact with numerous molecular targets in different cell types. The anti-atherogenic potential of curcumin consists in its capacity to lower blood cholesterol and TG in healthy subjects (80 mg/day for 4 weeks) [47]. It is reported that turmeric inhibits LDL oxidation in atherosclerotic rabbits and increases serum HDL-C levels in diabetic rats [48]. The hypolipidemic effect of curcumin is based on the inhibition of the intestinal cholesterol absorption and increased activity of cholesterol-7alpha-hydroxylase (CYP7A1), the rate-limiting enzyme in the synthesis of bile acids [48]. In addition, curcumin inhibits 3-hydroxy-3-methyl-glutaryl-coenzyme A (HMG-CoA) reductase. Administration of 0.02% *w*/*w* curcumin for 18 weeks to ldlr^-/-^ mice fed a high-cholesterol diet induces an inhibition of the hepatic TG accumulation by upregulation of peroxisome proliferator-activated receptors alpha (PPARα) and liver X receptor alpha (LXRα) expression [49]. PPARα is an important activator of fatty acid oxidation and inhibitor of hepatic fatty acid synthase (FAS) activity. LXRα regulates the gene expression of the key enzyme involved in cholesterol conversion to bile acid (CYP7A1), and increases expression of liver apolipoprotein A-I (apoA-I) and ATP-binding cassette A1 (ABCA1), which facilitates the HDL-mediated RCT [50]. It is known that an increase of 1 mg/dL in HDL-C level reduces coronary heart disease risk by 2–3%, and CVD mortality risk by 3.7–4.7% [51].

The antioxidant action of curcumin resides in the inhibition of ROS production by repressing the catalytic subunits p67phox, p47phox and p22phox of nicotinamide adenine dinucleotide phosphate (NADPH) oxidase [52,53]. In parallel, curcumin reduces ROS by upregulating the expression of endogenous antioxidant enzymes, such as SOD, catalase, GSH-Px and HO-1 [54,55]. Another interesting mechanism by which curcumin exerts antioxidant effects is the preservation of the mitochondrial redox potential [13] that has been evidenced in vivo in rat hearts subjected to ischemia-reperfusion. Curcumin pretreatment increases mitochondrial SOD activity and decreases mitochondrial H_2_O_2_ and malondialdehyde (MDA) levels [13]. HO-1 is an enzyme activated by oxidative stress which reduces inflammation by inhibiting the expression of endothelial adhesion molecules [56]. It was reported that curcumin can induce HO-1 in TNF-α-treated EA.hy926 cells in a dose-dependent manner and through activation of the transcription factor Nrf2. It also decreases ICAM-1 expression in a mouse model of lung injury [57,58]. As in EC, in vascular smooth muscle cells (SMC) curcumin activates Nrf2 which increases aldose reductase, an important enzyme that reduces oxidative stress in phosphatidylinositol 3-kinase (PI3K)/protein kinase B (Akt) and p38 mitogen-activated protein kinase (p38 MAPK)/c-Jun *N*-terminal kinase (JNK)-dependent manners [59]. These effects of curcumin on Nrf2 pathway have also been seen in human clinical trials involving patients with type 2 diabetes mellitus who each received an oral dose of curcumin of 500 mg/day for 15–30 days. This treatment induced an upregulation of Nrf2 in lymphocytes that controls other proteins, such as NAD(P)H quinone dehydrogenase 1 (NQO1), reduces inflammatory markers, and reduces plasma MDA levels [60]. In addition, curcumin supplementation of the diet increases NO bioavailability and inhibits the expression of pro-oxidative NADPH oxidase 2 (NOX-2) enzyme in rats [61]. Curcumin inhibits NF-κB and further decreases the expression of pro-inflammatory cytokines such as TNF-α, IL-1β and IL-6 both in vitro and in vivo [62].

There is growing interest in using curcumin to reduce vascular disease, in part due to its demonstrated anti-inflammatory effects. Using the in vitro model of TNF-α-activated EC, it was demonstrated that curcumin reduces the expression of VCAM-1, ICAM-1, E-selectin [63], fraktalkine and P-selectin [64], thereby inhibiting significantly monocyte adhesion through mechanisms involving the reduction of NADPH oxidase activation and consequently of the intracellular ROS production [64]. These results were confirmed in a recent study by Monfoulet et al., who demonstrated that curcumin pre-exposure reduces endothelial permeability and monocyte adhesion in both static and flow conditions [65]. In addition, most of the in vitro and in vivo studies confirm that curcumin administration determines the lowering of MCP-1 levels by downregulation of the MAPK and NF-κB signaling pathway [66,67,68]. The anti-inflammatory effects of curcumin on EC inflammatory markers were demonstrated also in vivo in various animal models. Tsai et al. evidenced the decrease of VCAM-1, ICAM-1 and CRP levels after curcumin supplementation to rats fed with a high-sucrose and high fat diet. The mechanisms that determine the improvement of the vascular function in this animal model involved an enhanced NO production and a reduction of the oxidative stress due to the increase of antioxidant enzyme activities [69]. SMC, an important component of the vascular wall, participates in the characteristic inflammatory process of atherosclerosis. In an in vitro study, Han et al. demonstrated that migration of human aortic SMC isolated from spontaneously hypertensive rats (SHR) or normal rats exposed to angiotensin II (AngII) was significantly inhibited by curcumin through the reduction of NLRP3/NF-κB signaling pathway [70]. The results were confirmed in vivo; it was demonstrated that administration of curcumin in SHR reduced intima-media thickness due to the inhibition of NF-κB and NLRP3 inflammasome and matrix metallopeptidase 9 (MMP-9) expression [70,71]. Yin et al. showed that curcumin inhibited caspase-1 activation and IL-1β secretion through suppressing lipopolysaccharide (LPS) priming and NLRP3 inflammasome in mouse bone marrow-derived macrophages, and confirmed these results in vivo in a model of high-fat diet-induced insulin resistance in wild-type C57BL/6 mice [72]. Interestingly, in an in vitro study, Chen et al. demonstrated that curcumin inhibits the M1 inflammatory phenotype of RAW264.7 macrophages as a result of the direct activation of the inhibitor protein κB-α (IkB-α) and polarizes the macrophages to become anti-inflammatory M2 phenotype through the activation of PPARγ [73]. Later on, the same group reported that curcumin dramatically reduced oxLDL-induced IL-1β, IL-6 and TNF-α cytokine production in M1 derived from M0 RAW264.7 macrophages [74].

In vivo studies demonstrated the benefic effects of curcumin to reduce the inflammatory burden present in the ischemia/reperfusion (I/R) in different organs by modulating the expression of different signaling pathways. In the brain tissue of rats after cerebral ischemia, curcumin decreased TNF-α and IL-6 levels via activation of SIRT-1 [75]; reduced TNF-α, IL-1β, IL-6, and high mobility group box 1 (HMGB1) by inhibition of the Janus kinase 2 (JAK2)/signal transducers and activator of transcription 3 (STAT3) signaling pathway [76]; and inhibited ICAM-1 and MMP-9 by downregulating NF-kB and elevating Nrf2 [77] or reduced TNF-α and IL-1β by inhibiting the TLR2/4/NF-kB signaling pathway [78]. It was reported that curcumin inhibits the activation of JNK and activator protein 1 (AP-1) transcription factor, and also the phosphorylation and degradation of IκB-α [79].

In a recent meta-analysis that proposed to evaluate the therapeutic effect of curcumin in mouse models of atherosclerosis, Lin et al. found that curcumin significantly decreases the aortic atherosclerotic lesion area, and the serum lipid levels (TC, TG and LDL-C) and inflammatory markers (TNF-α and IL-1β) [80]. In addition, Lin et al. highlighted the dose-response relation between curcumin and its protective effect on atherosclerosis, showing that the effect on decreasing the aortic lesion area is stronger in low and medium dosages (< 207 mg/kg BW/day) and weaker when the dose was more than 207 mg/kg BW/day, becoming pro-atherogenic when the dose reached 347 mg/kg BW/day [80]. Clinical trials focusing on curcumin effects in atherosclerosis progression gave dissimilar results, some of them evidencing that curcumin has no effect on risk factors of atherosclerosis [81,82], while others reporting an atheroprotective effect of curcumin by improvement of the lipidic profile in patients with metabolic syndrome, patients taking curcumin extract capsules (630 mg thrice daily) for 12 weeks [83]. In a pilot study, Panahi et al. demonstrated that administration of curcuminoids (500mg/day, for 4 weeks) to subjects with pulmonary problems reduces the inflammatory mediators’ expression: IL-6, IL-8, TNF-α, transforming growth factor β (TGF-β), high sensitivity C-reactive protein (hsCRP) and MCP-1 [84]. There are many studies supporting the anti-inflammatory properties of curcumin; however, of great importance is the establishment of a proper treatment with regard to the dose and time of administration.

To improve curcumin stability in vivo, different types of nanocarriers have been described for encapsulation. Thus, lipid nanoemulsions loaded with curcumin and functionalized with a cell-penetrating peptide were better taken up and internalized by human EC compared to the non-functionalized lipid nanoemulsions. This formulation of curcumin demonstrated anti-inflammatory effects by reducing the monocytes adhesion to TNF-α activated human EC [85].

In the last decade, published data evidenced that some phenolic compounds are able to perform a fine-tuning regulation of the mechanisms underlying oxidative and inflammatory stress by modulating the expression of epigenetic factors associated with RNA function, such as ncRNAs. Accordingly, recent in vitro and in vivo experiments showed that specific miRNAs mediate the molecular mechanisms affected by curcumin. Thus, treatment of murine Raw264.7 and human THP-1 macrophages with curcumin significantly reduced miR-155 expression through the modulation of PI3K/Akt pathway [86]. In the same report, septic mice obtained by LPS intraperitoneal injection were treated orally with curcumin, and a significant reduction of miR-155 expression and Akt phosphorylation in the liver and kidney was observed [86]. In a recent in vivo study of Zhang et al., mice with peripheral arterial disease (PAD) and a normal glycaemic profile were treated with curcumin [87]. They reported that curcumin treatment improved perfusion recovery, increased capillary density and increased miR-93 expression in ischemic muscle tissue. Moreover, in cultured EC under simulated ischemia, curcumin improved cell viability and enhanced tube formation. These data proved that curcumin may have beneficial effects in non-diabetic PAD by improving angiogenesis, which may have been achieved partially via the promotion of miR-93 expression [87]. In another in vivo experiment, after curcumin administration, C57BL/6 mice were subjected to left anterior descending coronary artery occlusion [88]. Geng et al. showed that curcumin administration significantly reduced the infarct size compared with control animals, increased miR-7a/b expression and downregulated the expression of transcription factor specific protein 1 (SP1). In hypoxia-induced mouse cardiac myocytes (MCM), curcumin led to the decrease of cell apoptosis. The authors suggested that curcumin pretreatment protected against hypoxia-induced MCM apoptosis through the upregulation of miR-7a/b and the downregulation of SP1 expression [88].

Curcumin was reported to modulate some miRNAs that are dysregulated in diabetes. In a study conducted by Tian et al., miR-17-5p was proven to stimulate adipogenic differentiation of mouse 3T3-L1 cells [89]. This gene encodes a key Wnt signaling pathway effector, and its human homologue transcription factor 7 like-2 (TCF7L2) is a highly diabetes risk gene. After treatment with curcumin, a decrease of miR-17-5p expression was observed, together with an increase of its target gene, Tcf7l2, in 3T3-L1 adipocyte cells. The authors also reported an elevation of miR-17-5p expression in mouse epididymal fat tissue in response to high fat diet. The authors suggested that miR-17-5p is among the central switches of adipogenic differentiation, activating adipogenesis via repressing the Wnt signaling pathway effector Tcf7l2, and its own expression is nutritionally regulated by curcumin [89].

*Caffeic acid* (3,4-dihydroxycinnamic acid) (Figure 1) is the major dietary hydroxycinnamic acid and is found in food, mainly as caffeic acid phenethyl ester (CAPE) or chlorogenic acid (5-O-caffeoylquinic acid), which results from its conjugation with quinic acid. The chlorogenic acid is one of the most widely consumed polyphenols, being present in many fruits (blueberries, apples, pears), vegetables (lettuce, potatoes, eggplants), and beverages, including coffee (caffeinated or decaffeinated), wine and tea. Regular consumption of coffee results in the ingestion of 0.5–1 g of chlorogenic acid and 250–500 mg of caffeic acid/day [90,91,92]. CAPE has poor bioavailability attributed to its low aqueous solubility, and in the plasma undergoes rapid hydrolysis to caffeic acid as the major metabolite. To overcome the poor bioavailability of CAPE, different formulations such as chemical modifications or microencapsulation in cyclodextrins were developed with success, the aqueous solubility of CAPE was notably increased [93]. The low bioavailability of caffeic acid (14.7%) is due to the low intestinal absorption and low permeability across the intestinal cells [94]. No formulations of caffeic acid to be used for CVD treatment have been developed at present.

CAPE has been reported to have antioxidant and anti-inflammatory properties [95]. It was demonstrated that CAPE induces the expression of redox-sensitive HO-1 [96] through activation of the Kelch-like ECH-associated protein 1 (Keap1)/Nrf2/antioxidant response element (ARE) pathway [97], and consequently generates the transcription and translation of detoxifying and antioxidant phase II cytoprotective enzymes. Nrf2 is bound to Keap1 in the cytoplasm before activation, and once inducers react with the sulfhydryl groups of Keap1, Nrf2 is released and eventually translocated into the nucleus, where it binds to and activates ARE, which acts as a promoter/enhancer regulating the genes of the mentioned antioxidant enzymes [98]. The Nrf2/ARE pathway can be also activated through Nrf2 phosphorylation by PI3K/Akt, extracellular signal-regulated kinases (ERK) or MAPK [97]. In addition, administration of CAPE to rats stimulates PON1 expression in the lung exposed to inflammatory stimuli [99].

The benefic effect of caffeic acid on inflammatory stress is not very well documented. It was reported that caffeic acid can reduce monocyte adhesion to TNF-α-activated human umbilical vein EC (HUVECs) by reducing the expression of VCAM-1, ICAM-1, E-selectin and MCP-1. These beneficial effects were attributed to the reduction of NF-κB p65 translocation from cytosol to nucleus, thereby decreasing the formation of NF-κB–DNA complex [100]. A relatively recent in vitro study confirmed these results and added new data, demonstrating that caffeic acid exerts anti-inflammatory effects on HUVEC exposed to glycated LDL (gLDL) by reducing the secretion of CRP, VCAM-1 and MCP-1. The downregulation of all these pro-inflammatory molecules is possible due to the inhibition of the receptor for advanced glycation end products (RAGE) expression and diminution of the oxidative stress (by reducing NOX4 and p22phox subunits of NADPH oxidase) and of the endoplasmic reticulum stress [101].

In vivo, it was shown that the caffeic acid reduces the plasma TNF-α, IL-6 and IL-8 in rats receiving a high-fructose diet by decreasing the oxidative stress due to restoring of the antioxidant enzymes concentration (SOD, catalase, GSH-Px, glutathione reductase and glucose 6-phosphate dehydrogenase) [102]. In a very interesting in silico study, the caffeic acid was identified as a potential therapeutic agent having anti-inflammatory potential due to its interactions with cyclooxygenase-1 (COX-1) and 2 (COX-2), coagulation factor Xa (FXa) and integrin αIIbβ3 proteins, which are directly or indirectly participants in the thrombosis pathways [103].

Another molecular mechanism associated with the positive effects of caffeic acid in ameliorating the lipid metabolism and oxidative and inflammatory stress is the regulation of epigenetic factors, in particular, miRNAs. Murase et al. showed that in vitro treatment of Hepa1-6 hepatocytes with coffee polyphenols significantly increased cellular miR-122 expression, and reduced sterol regulatory element-binding transcription factor 1c (SREBP-1c) expression [104]. Using high-fat diet/streptozotocin-induced diabetic rats, Matboli et al. showed that caffeic acid intake induced improvement in albumin excretion, blood glucose, reduced renal mesangial matrix extension with increased vacuolation and reappearance of autophagosomes [105]. Additionally, they demonstrated that caffeic acid treatment stimulates autophagy genes with simultaneous reduction in their epigenetic regulators: miR-133b, miR-342 and miR-30a. These data suggest that caffeic acid can modulate the autophagy pathway through inhibition of autophagy regulatory miRNAs that could explain its curative properties against diabetic kidney disease [105].

### 2.2. Stilbenes

**Figure 2:**
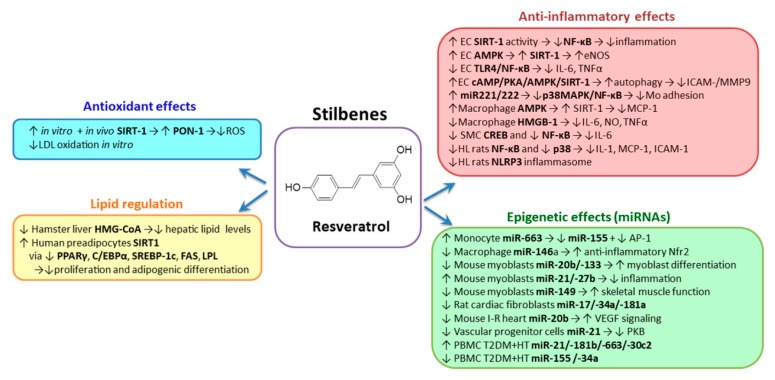
Resveratrol’s chemical structure and its demonstrated effects of improving cardiovascular disease outcomes.

*Resveratrol* (trans-3,5,4′-trihydroxystilbene) has a C6–C2–C6 structure containing three hydroxyl groups, which functions as a UV protectant of plants and defender against pathogenic infections (Figure 2). The food sources of resveratrol (RSV) are red wine, grapes, peanuts, passion fruit, white tea, plums and raspberries [106]. Oral ingestion of RSV is the most feasible route of administration, but the compound has low water solubility (~30 mg/L); it is rapidly metabolized, and consequently has a poor bioavailability. A slight increase in RSV solubility considerably enhances its bioavailability [107]. After ingestion, RSV is absorbed in the small intestine and then released into the bloodstream where it can bind to albumin and lipoproteins that further deliver RSV to the cells of the peripheral tissue. RSV is well tolerated and its plasma concentration depends on the dose consumed, but not in a linear relation. High oral doses (1 g/kg) may generate side effects (nausea, abdominal pain) [107]. Thus, improving RSV bioavailability will straighten its potential as a therapeutic agent. Recently the researchers have tried to increase RSV bioavailability by nanoencapsulation in lipid nanocarriers or liposomes, nanoemulsions, micelles, insertion into polymeric particles, solid dispersions and nanocrystals [108]. The results are promising, but further studies are needed to improve these methodological approaches and to compare effects of the most valuable strategies in the same trial.

The lipid-lowering action of RSV is based on its capacity to reduce the level and activity of HMG-CoA reductase that has been demonstrated in the livers of hamsters with diet-induced dyslipidaemia [109]. Studies conducted in humans are controversial, some of them evidencing a reduction of LDL-C and an increase of HDL-C [110,111], and others showing no effect of RSV on plasma lipid profile [112]. RSV has important effects on adipose tissue due to its ability to inhibit differentiation of preadipocytes and stimulation of lipolysis. Thus, RSV inhibits proliferation and adipogenic differentiation of human preadipocytes by a SIRT1-dependent mechanism and in the 3T3 cells downregulate the expression of PPARγ, CCAAT-enhancer-binding proteins α (C/EBPα), SREBP-1c, FAS and lipoprotein lipase (LPL), which are important regulators of the lipolysis [113]. RSV has protective effects in terms of CVD risk due to its capacity to improve PON1 activity. Experiments performed in vitro and in vivo evidenced the positive effects of quercetin/RSV on PON1 activity [95,114,115,116]. The mechanisms responsible for this effect might be the activation of SIRT-1, which is known as activator of LXR, important regulator of PON1 gene [117]. The antioxidant potential of RSV was evidenced by inhibiting macrophages-induced in vitro oxidation of LDL [118]. The ability of RSV to exert antioxidant effects in humans is still under investigation.

Numerous studies reveal that the benefic effects of RSV are due to its potential to activate several important anti-inflammatory targets [119], although the exact mechanisms of action have not been clearly elucidated [120]. It was shown that the main target of RSV is SIRT-1, and its binding determines modifications of the SIRT-1 structure that enhances the binding of SIRT-1 to its substrates [121]. An important substrate of SIRT-1 is p65 of NF-κB (RelA) [122], the key transcription factor involved in regulation of inflammatory cytokines [123]. SIRT-1 activation by RSV determines the inhibition of RelA acetylation, which in turn decreases NF-κB expression [124]. In addition, RSV inhibits p300 expression and promotes the IκB-α degradation [125]. Another molecular target of RSV is AMP-activated protein kinase (AMPK), a protein that controls the activity of SIRT-1 by regulating the available cellular levels of NAD^+^ [126]. Beside SIRT-1, AMPK is known to activate eNOS in EC. A recent clinical trial involving primary hypertensive patients evidenced that addition of a micronized formulation of RSV to standard antihypertensive therapy is sufficient to normalize the blood pressure, without additional antihypertensive drugs [127]. Other targets of RSV were identified by different other groups: TLR4 [128,129], miR-221/222 [130] and p38 MAPK [131,132].

RSV induces the decrease of endothelial activation and vascular inflammation, and improves the endothelial function. It was demonstrated that RSV determines the decrease of IL-6 and TNF-α via the TLR4/myeloid differentiation primary response gene 88 (MyD88)/NF-κB signal transduction pathway in HUVECs exposed to LPS [128]. In addition, it was reported that pre-incubation with RSV reduced the TNF-α-induced ICAM-1 secretion, as well as the intracellular expression of ICAM-1 and MMP-9 in EC by inducing autophagy, mediated in part through the activation of the cAMP/protein kinase A (PKA)/AMPK/SIRT-1 signaling pathway [133]. Liu et al. demonstrated that RSV decreases ICAM-1 expression and monocyte adhesion to TNF-α-exposed HUVECs by stimulating miR-221/-222 production, which determines p38 MAPK/NF-κB inhibition [130].

In THP-1 human macrophages stimulated with LPS, RSV pretreatment inhibited foam cells formation and reduced MCP-1 secretion, while increasing SIRT-1 and AMPK [134]. RSV significantly reduced the levels of secreted IL-6, NO and TNF-α in RAW264.7 cells exposed to LPS by attenuating HMGB-1 expression [135]. Other mechanisms of RSV action in LPS-exposed macrophages involve the attenuation of TLR4- TNF receptor-associated factor 6 (TRAF6), MAPK and Akt pathways [136].

The anti-inflammatory properties of RSV were demonstrated also in SMC. Inanaga et al. showed that RSV attenuates Ang II-induced IL-6 protein in the supernatant of vascular SMC in a dose-dependent manner. These effects were attributed to the ability of RSV to reduce the activity of cAMP-response element-binding protein (CREB) and NF-κB, two transcription factors which are critical for Ang II-induced IL-6 gene expression [137]. In addition, Zhang et al. demonstrated that RSV reduced the proliferation of vascular SMC exposed to Ang II by inhibiting ERK1/2 phosphorylation and NF-ĸB transcriptional activity [138].

The anti-inflammatory properties of RSV were demonstrated also in vivo. Using hyperlipidemic rats, Deng et al. demonstrated that RSV decreases the serum levels of IL-1β and reduces MCP-1, ICAM-1, p65 NF-κB and p38 MAPK mRNA and protein expression in the thoracic aortas samples [139]. In addition, NLRP3 inflammasome oligomerization was also decreased in the aortic tissue, in parallel with the upregulation of SIRT-1 expression [139]. Interestingly, Chang and colleagues previously demonstrated that RSV reduces inflammation, such as aortic macrophage infiltration and NF-κB expression in apoE-deficient mice fed with a high-cholesterol diet [140]. Using the model of an I/R-injured rat, Cong et al. demonstrated that RSV reduced the myocardial infarct area, in parallel with a reduction of serum and myocardial TNF-α levels through a mechanism dependent on NO production [141]. Li et al. confirmed the previous in vivo study, demonstrating that RSV significantly reduces myocardial infarct size and myocardial apoptosis, serum and myocardial TNF-α production by a mechanism dependent on TLR4/NF-κB attenuation and NO production [142].

The results concerning anti-inflammatory potential of RSV in humans were contradictory, some studies evidencing a positive effect in healthy people [143], others showing no effects in postmenopausal women [144]. The contrasting results suggest that in order to obtain beneficial effects, the dose and the way of administration have to be carefully analyzed. It was suggested that a moderate (>450 mg) continuous intake is better than a single, higher dose administration [145].

Recent in vitro and in vivo experiments proved that RSV positively regulates the mechanisms underlying oxidative and inflammatory stress by modulating the expression of a set of specific miRNAs. Tili et al. showed that RSV upregulates miR-663 in human THP-1 and circulating monocytes, this miRNA being proven as anti-inflammatory by inducing the decrease of AP-1 transcriptional activity [146]. Moreover, they showed that RSV impairs AP-1 upregulation induced by LPS at least in part by targeting JunB and JunD transcripts. In contrast, RSV impairs the LPS-induced upregulation of pro-inflammatory miR-155 in a manner dependent of increasing miR-663 levels [147]. These data suggest the potential modulation of miR-663 levels to stimulate the anti-inflammatory effects of RSV in metabolic disorders associated with elevated levels of miR-155. Since many in vitro experiments use high concentrations of phenolic compounds and do not reproduce their physiological in vivo plasma levels, Bigagli et al. incubated RAW264.7 macrophages with corresponding plasma physiological concentrations of RSV, hydroxytyrosol and oleuropein [148]. They showed that only RSV and hydroxytyrosol (at 10 μM) decreased miR-146a, which is known to target Nrf2 responsible for inhibiting pro-inflammatory mediators. In addition, the authors showed that Nrf2 was increased by RSV and hydroxytyrosol after in vitro stimulation of murine macrophages with LPS [148]. Lançon et al. reported that of 26 miRNAs were increased (miR-21 and miR-27b) in prevalence by RSV in mouse C2C12 skeletal myoblasts, while other 20 miRNAs (miR-20b and miR-133, a muscle-specific miRNA known to target genes involved in myoblast differentiation) were downregulated [149,150]. Additionally, miR-149 was downregulated by RSV, this miRNA having potential role in the regulation of skeletal muscle functionality. Recently, Zhang et al. demonstrated that RSV can inhibit in vitro the TGF-β1-induced proliferation of rat cardiac fibroblasts (CF) and collagen secretion [151]. RSV also decreased miR-17, miR-34a and miR-181a in TGF-β1-treated CF. The authors suggested that the inhibitory effect of RSV is mediated by the downregulation of miR-17 and the regulation of Smad7 [151].

In an in vivo study, Mukhopadhyay et al. reported the cardioprotective effect of RSV and proposed a RSV-induced miRNAs profile in a rat I/R model [152]. They reported that RSV significantly downregulated miR-20b, which might modulate vascular endothelial growth factor (VEGF) signaling. This downregulation of miR-20b was proposed to be linked with the potent anti-angiogenic action of RSV in the ischemic myocardium and with the synergic effects of RSV and γ-tocotrienol. An elegant and complex study performed by Campagnolo et al. showed that RSV can induce the expression of endothelial markers, such as CD31, VE-cadherin and eNOS in vascular resident progenitor cells and embryonic stem cells [153]. They also demonstrated that RSV significantly reduced miR-21 expression in these cells, which in turn diminished protein kinase B (PKB) phosphorylation. This signaling cascade reduced the nuclear β-catenin, inducing endothelial marker expression and increasing tube-like formation by progenitor cells. Additionally, the authors showed that vascular progenitor cells treated ex vivo with RSV produced better endothelialization of the decellularized vessels. Moreover, they demonstrated that RSV-enriched diet reduces lesion formation in a mouse model of vessel graft [153].

Tomé-Carneiro et al. performed a randomized placebo-controlled study with type-2 diabetic and hypertensive men, who received capsules containing either placebo (maltodextrin), grape extract (laking RSV) (GE) or grape extract with over 8 mg of RSV (GE-RES) during one year [154]. Their results show that supplementation with GE or GE-RES did not affect body weight, blood pressure, glucose, HbA1c or lipids, beyond the values regulated by gold standard medication in these patients. They also found molecular changes in peripheral blood mononuclear cells (PBMC), evidenced by the significantly reduced expression of the pro-inflammatory cytokines—macrophage inflammatory protein 1α (MIP1α), CCL3, IL-1β and TNF-α—and increased expression of transcriptional repressor leucine-rich repeat flightless-interacting protein 1 (LRRFIP-1) in PBMC from patients taking the GE-RES extract for 12 months [154]. Additionally, a GE-RES treatment-associated modulation of miRNAs involved in the inflammatory response was noticed, demonstrated by the increase of a set of miRNAs miR-21, miR-181b, miR-663 and miR-30c2, together with a decrease of miR-155 and miR-34a in PBMC after GE-RES treatment, as compared to the control group. These data provide evidence for the in vivo modulation of inflammatory miRNAs in PBMC by RSV in circulating immune cells of diabetic hypertensive medicated patients and support a beneficial immunomodulatory effect in these patients [154].

### 2.3. Flavonoids Group

*Flavonoids* (including flavonols, flavones, flavanones, flavanols, isoflavones and anthocyanidins) exert multiple beneficial effects. The food sources of flavonoids are berries, black tea, celery, citrus fruits, green tea, olives, onions, oregano, purple grapes, purple grape juice, soybean, soy products, vegetables, whole wheat and wine [155].

#### 2.3.1. Flavonols

**Figure 3:**
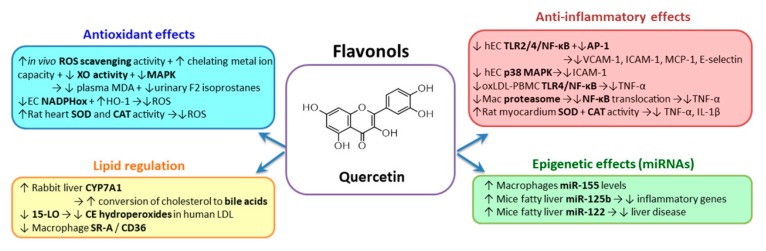
Quercetin chemical structure and beneficial effects in the context of cardiovascular diseases.

*Quercetin* (3,3′,4′,5,7-pentahydroxylflavone) (Figure 3) is administered as quercetin-3-glucoside (isoquercetin) which is hydrolyzed to quercetin in the small intestine, rapidly absorbed and then transferred into the blood. Vegetables that are important sources of quercetin are apples, grapes, berries, black tea, green tea, red onions, kale, leeks, broccoli, apricots, pepper, red wine and tomatoes [155]. A very recent study shows that quercetin alters the gut microbiota and reduces the atherogenic lipids, such as cholesterol and lysophosphatidic acids, all these effects being associated with the diminution of atherosclerotic lesions area [156]. Unlike most phenolic compounds, quercetin has a relatively high bioavailability. It is absorbed in the small intestine, then undergoes different transformations in the small intestine, colon, liver and kidney. Quercetin that is not intestinally absorbed is further subjected to colon microflora metabolization. Ingested quercetin is rapidly eliminated as metabolites through feces and urine. The bioavailability of quercetin orally administered to humans was estimated at ~45% with 3.3–5.7% of the dose found in the urine and 0.2–4.6% in the feces [113,157].

Quercetin is efficient at stimulating cytochrome P450 and CYP7A1 levels, and the conversion of cholesterol to bile acids in the liver of rabbits fed with a high fat diet [158]. By inhibition of 15-lipoxygenase, quercetin and its monoglucoside derivatives inhibits cholesteryl ester hydroperoxides formation in human LDL [158]. Antioxidant quercetin metabolites like quercetin-3-glucuronide (Q3GA) are taken-up by the human macrophages present in the intima and convert them to methylated derivatives, which suppress the gene expression of scavenger receptors SR-A and CD36 [159].

The data supporting the antioxidant potential of quercetin are contradictory. Thus, administration of quercetin at 10 mg/kg bodyweight for 13 weeks was shown to downregulate NADPH oxidase and increase eNOS activity, improving the endothelial function in hypertensive male rats [160], while a higher dose (1.5 g quercetin/kg diet for 5–11 weeks) was not associated with a reduced risk of developing CVD in hypertensive rats [161]. In such situation it is necessary to test the compound in the same experimental model, but at several concentrations to determine the optimum and identify the harmful one. In vivo, the antioxidant potential of quercetin was expressed as lower levels of urinary isoprostane F2 and plasma MDA, that could be due to the ability of quercetin to scavenge ROS, chelate metal ions, reduce xanthine oxidase activity and to inhibit the MAPK pathway [162,163]. The mechanism by which quercetin increases eNOS activity in a dose-dependent manner involves the phosphorylation on Ser1179 by cAMP/PKA pathway [164]. The antioxidant potential of quercetin is also reflected in the decrease of the oxidation levels of LDL [165]. In addition, quercetin reduces the activities of SIRT-1 and AMPK, upregulates HO-1 and decreases the expression of oxLDL-induced NOX2 and NOX4 in human EC [166]. Kaempferol (50 or 100 mg/kg for 4 weeks), another member of the flavonols group, reduces atherosclerotic lesions area in apoE-deficient mice through mechanisms involving reduction of the aortic ROS production and osteopontin downregulation [167]. Furthermore, kaempferol diminishes oxLDL-enhanced apoptosis of EC based on the upregulation of autophagy by inhibition of PI3K/Akt/mammalian target of rapamycin (mTOR) pathway [168].

Extensive studies using in vitro or in vivo models clearly indicate that quercetin manifests anti-atherosclerotic effects, in part due to its anti-inflammatory properties. In vitro studies demonstrate that quercetin reduces the expression of VCAM-1, ICAM-1, E-selectin or MCP-1 in cultured human EC exposed to different pro-inflammatory stimuli. The molecular mechanisms involve modulation of TLR2/4/NF-kB and AP-1 transcription factor. In addition, it was demonstrated that quercetin reduces ICAM-1 in EC exposed to uremic media by downregulating p38 MAPK [169,170,171,172]. Anti-inflammatory properties of quercetin were demonstrated also in monocytes/macrophages. It was demonstrated that TNF-α released by oxLDL-exposed human PBMC is reduced by quercetin through modulation of TLR/NF-κB signaling pathway [170]. In LPS-stimulated macrophages isolated from C57BL/6 and BALB/c mice, quercetin reduces the secretion of TNF-α and NO produced by the inducible NO synthase (iNOS) by a mechanism involving the inhibition of proteasome, which determines a diminished proteolytic degradation of phospho-IκB protein, resulting in the decreased translocation of activated NF-κB to the nucleus [173].

The decrease of the atherosclerotic lesions was not associated with the improvement of the lipid profile, but with the decreased inflammatory stress, measured as decreased IL-1 receptor, IKK and STAT3 [169,174]. In the rat model of acute myocardial infarction, quercetin administration determined the reduction of TNF-α, IL-1β expression in the myocardial tissue, in parallel with an increase of the antioxidant SOD and catalase activities [175]. Recently, a few clinical studies regarding the anti-inflammatory effects of quercetin in human subjects were published, but the results are controversial. A double-blind randomized clinical trial designed to measure the anti-inflammatory effects of quercetin (500 mg) administrated for 10 weeks to women with type 2 diabetes reported no differences between groups [176]. Another study conducted on stable angina patients receiving 120 mg/day quercetin for two months showed a statistically significant reduction of IL-1β levels and attenuated TNF-α and IL-10 levels in treated patients’ plasma, and decreased transcriptional activity of NF-kB in PBMC [177]. Several studies were done to evaluate the safety of quercetin administration to humans [178]. The conclusion of these studies was that up to 5000 mg/day quercetin supplementation for four weeks does not cause adverse effects.

In addition, quercetin can exert its positive effects on the regulation of oxidative and inflammatory stress by modulating the expression of specific miRNAs. Thus, Boesch-Saadatmandi et al. reported that quercetin and isorhamnetin upregulated miR-155 levels in LPS-activated macrophages, but quercetin metabolites, such as quercetin-3-glucoronide, did not modify miR-155 expression [179]. An in vivo study of the same group showed that addition of quercetin to high-fat diet fed C57BL/6J mice significantly increases hepatic expression of miR-125b, a negative regulator of inflammatory genes, and miR-122, known to be involved in lipid metabolism and pathogenesis of liver diseases [180]. These data suggest that miRNAs could represent potential targets of quercetin in the CVD prevention or treatment.

#### 2.3.2. Flavones

**Figure 4:**
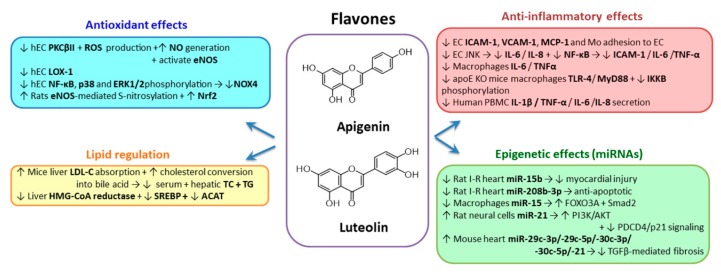
Antioxidant, anti-inflammatory, lipid-lowering and epigenetic mechanisms to improve cardiovascular diseases outcomes demonstrated by apigenin and luteolin.

*Apigenin* (4′,5,7-trihydroxy-flavone) is one of the major monomeric flavonoids existing in the diet and is found in a glycosylated form, with the tricyclic core structure linked to a sugar moiety through hydroxyl groups (O-glycosides) or directly to carbon (C-glycosides) (Figure 4). Apigenin is present in fresh parsley, vine spinach, celery seed, green celery heart, chinese celery, dried oregano, chamomile tea, red and white sorghum, rutabagas, oranges, kumquats, onions, wheat sprouts, tea and cilantro [181]. Apigenin-glycosides can be hydrolyzed in vivo into apigenin or chrysin. Oral bioavailability of apigenin is relatively low due to its poor solubility and because the main part of the ingested apigenin is either excreted unabsorbed or is rapidly metabolized after absorption. In vivo, after ingestion, apigenin is subjected to sulfation and glucuronidation, the absorbed apigenin being present in tissues (mainly hepatic and small intestin) as glucuronide, sulfate conjugates or luteolin [182].

Apigenin decreased the serum and hepatic levels of TC and TG in hyperlipidemic mice by promoting liver LDL-C absorption and increasing the conversion of hepatic cholesterol into bile acid [183]. In addition, apigenin markedly lowered the levels of hepatic enzymes involved in the synthesis of TG and cholesterol esters in HFD-induced obese mice, thereby ameliorating hepatic steatosis [184].

Data supporting the protective role of apigenin on the vascular wall in diabetes reveal the impeding of the endothelial dysfunction by inhibition of high-glucose-mediated protein kinase CβII (PKCβII) upregulation and ROS production, while stimulating NO generation [185]. The molecular mechanism of eNOS activation by apigenin involves continuous eNOS Ser1179 phosphorylation by the PI3K/Akt pathways [186]. In addition, apigenin inhibits oxLDL receptor 1 (LOX-1) expression after stimulation of EC with high glucose and TNF-α, LOX-1 being an important receptor involved in the uptake of modified lipoproteins and atherosclerotic plaque progression [187].

The anti-inflammatory properties of apigenin have been investigated in studies conducted in vitro and in vivo. Using cultured human EC activated with di-(2-ethylhexyl) phthalate, Wang et al. showed that apigenin suppressed the expression of ICAM-1 and inhibited THP-1 monocytic cells adhesion to HUVECs [188]. In addition, a dose-dependent inhibition of endothelial IL-6 and IL-8 expression was observed, and these inhibitory effects of apigenin are mediated by the JNK pathway, but not by IκBα/NF-κB or ERK pathways [188]. Apigenin inhibited NF-κB activation and ICAM-1 expression in EC exposed to palmitic acid [189]. In EC exposed to uremic plasma, p38 MAPK is another molecular target for apigenin [172]. Regarding the effect of apigenin on cytokine secretion, it has been shown that apigenin reduced IL-6 and TNF-α secretion in LPS-stimulated RAW 264.7 macrophages [190] and decreased TNF-α release in the media of LPS-activated macrophages [191]. Apigenin in LPS-exposed macrophages reduces TLR-4, MyD88 and phosphorylation of IκKB levels through nuclear NF-κB p65 signaling pathway [192]. Ren et al. confirmed these results in vivo, demonstrating that in LPS-challenged apoE deficient mice, treatment with apigenin determined the reduction of TLR-4 and NF-κB p65 levels and lessened the macrophages and SMC number in atherosclerotic regions [192]. Apigenin inhibited the expression of VCAM-1 and IκKB kinase and prevented the adhesion of U937 monocytes to EC exposed to high-glucose (30 mM) concentrations [193]. The beneficial effects of intra-gastric administration of apigenin to type 2 diabetic (T2D) rats were expressed as decreases of the blood glucose concentration and ICAM-1 levels and improved impaired glucose tolerance [189].

*Luteolin* (3′,4′,5,7-tetrahydroxyflavone) (Figure 4) is a member of the flavones family and is found in carrots, cabbage, artichokes, tea, celery and apples [194]. Luteolin and its glucosides are absorbed quickly in the intestine. The time of maximum blood concentration is under one hour, and the maximum plasma concentration is of 1–100 µmol/L, depending on the dose ingested and the type of food consumed; the purer the luteolin used, the faster the absorption [195].

Luteolin exerts lipid-lowering effects due to the interaction with HMG-CoA reductase, the SREBPs and acyl-CoA cholesterol acyltransferase (ACAT) in the liver [3]. In all studies available, no major side effects have been detected, confirming the good tolerability and safety of artichoke extract, but long-term safety studies are needed [196,197]. There are no data about luteolin effects on the EC function or atherosclerotic plaque. Luteolin was shown to impede TNF-α–induced NOX4, which generates a decrease of ROS production in human EC. The mechanism involves the inhibition of the TNF-α–induced transcriptional activity of NF-κB, p38 and ERK1/2phosphorylation [198]. Recently, it was shown that luteolin (100 mg/kg/d) reduces cardiac I/R injury by enhancing eNOS-mediated S-nitrosylation and Nrf2 redox function in diabetic rats [199].

The effects of luteolin on monocyte adhesion to EC, a key event in triggering vascular inflammation, were evaluated in a few studies. Jia et al. demonstrated that physiological concentrations (0.5 µM) of luteolin suppress TNF-α-induced expression of MCP-1, VCAM-1 and ICAM-1 [200]. In addition, luteolin inhibited endothelial NF-κB transcriptional activity, IκB-α degradation and IκB-β expression, and thus reduced NF-κB p65 nuclear translocation. The in vitro results were confirmed in vivo using C57BL/6 mice fed with luteolin diet supplementation. The authors show that luteolin suppresses TNF-α-induced MCP-1 and soluble ICAM-1 in the plasma, as well as VCAM-1 in the aorta of mice [200]. In a recent study, Zhang et al. demonstrated that a combination of luteolin (0.5 µM) and curcumin (1 µM) inhibits, synergistically, VCAM-1 and MCP-1, and the subsequent monocyte adhesion to EC exposed to TNF-α by suppressing NF-kB translocation [201]. These results were confirmed in vivo in C57BL/6 mice that received luteolin and curcumin [201]. In human monocytes exposed to high glucose concentrations, luteolin significantly reduced IL-6 and TNF-α by inhibiting NF-κB activity [202]. Recently, it was demonstrated that luteolin effectively suppresses IL-1β, TNF-α, IL-6 and IL-8 secretion from PBMC from healthy donors incubated with LPS [203], confirming reports showing that luteolin reduces IL-6 and TNF-α secretion in LPS-stimulated RAW 264.7 macrophages [190]. Hong et al. evaluated the anti-inflammatory effects of luteolin in renal I/R injury in Sprague–Dawley rats and demonstrated that this natural compound attenuated serum and renal TNF-α, IL-1β and IL-6, and the renal HMGB1 and NF-κB expression levels in I/R rats [204]. In addition, luteolin significantly reduced the endoplasmic reticulum stress and renal cell apoptosis caused by renal I/R injury [204]. Recently, Ding et al. demonstrated that luteolin attenuates atherosclerosis in high-fat fed apoE-/- mice by alleviating inflammation through inhibition of signal transducer and STAT3 [205].

Recent studies evidenced that apigenin and luteolin modulate the expression of certain epigenetic factors, in particular miRNAs, that constitute fine regulators of the oxidative and inflammatory stress. Thus, an in vivo study by Wang et al. shows that the increased cardiac miR-15b expression observed during myocardial I/R injury in rats correlates with the decreased expression of JAK2 and activity of JAK2/STAT3 pathway, with augmented myocardial apoptosis and ROS production, and aggravated heart injury [206]. Apigenin treatment of I/R rats downregulates miR-15b expression in the heart, improves the altered mechanisms and alleviates myocardial I/R injury [206]. In an in vivo report, Bian et al. showed that luteolin pretreatment induces anti-apoptotic effects by decreasing miR-208b-3p expression in myocardial tissue of I/R rats [207]. Arango et al. performed a high-throughput PCR screening of 312 miRNAs in RAW 264.7 murine macrophages and evidenced that apigenin reduces LPS-induced miR-155 expression by transcriptional regulation [208]. They further demonstrated that apigenin-reduced expression of miR-155 led to the increase of the anti-inflammatory mediators forkhead box O3a (FOXO3A) and α smooth-muscle-actin (α-SMA) and MAD-related protein 2 (Smad2) in LPS-treated murine macrophages. Arango et al. also demonstrated that in vivo apigenin or a celery-based apigenin-rich diet reduced LPS-induced expression of miR-155 and decreased TNF-α in lungs from LPS-treated mice, thereby diminishing the inflammatory process [208].

Zhang et al. reported recently that low concentrations of luteolin presented protective effects on H_2_O_2_-induced ischemic cerebrovascular disease cell viability loss, proliferation inhibition, ROS generation, oxidative stress increase and apoptosis, together with an increase of miR-21expression level [209]. Furthermore, they showed that luteolin alleviated H_2_O_2_-induced inactivation of PI3K/Akt pathway and activated programmed cell death protein 4 (PDCD4)/p21 pathway in PC-12 cells by upregulating miR-21 [209]. Ning et al. showed that pretreatment and post-treatment with luteolin-7-diglucuronide (L7DG), a naturally occurring flavonoid glycoside found in leaves of basil or *Verbena officinalis*, significantly attenuated isoproterenol-induced myocardial injury and fibrosis in mice [210]. Furthermore, L7DG pretreatment blocked isoproterenol-stimulated expression of genes encoding the enzymatic subunits of NADPH oxidase (Cyba, Cybb, Ncf1, Ncf4 and Rac2). In addition, the authors showed that L7DG pretreatment almost reversed isoproterenol-altered expression of miRNAs which were cross-talking with TGF-β-mediated fibrosis, including miR-29c-3p, miR-29c-5p, miR-30c-3p, miR-30c-5p and miR-21 [210].

#### 2.3.3. Flavanones

**Figure 5:**
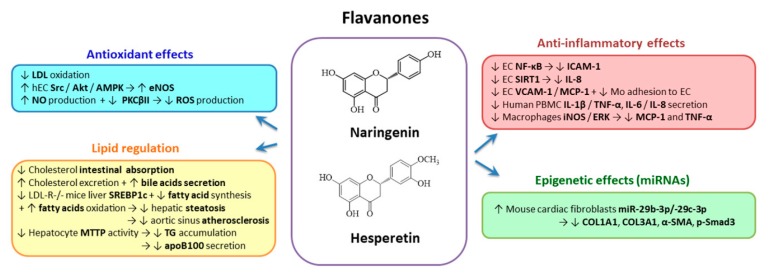
Chemical structure and cardioprotective mechanisms of action demonstrated by naringenin and hesperetin in experimental and clinical studies.

*Naringenin* (4,5,7-trihydroxy-flavanone) and *hesperetin* (3,5,7-trihydroxy-4′-methoxyflavanone) (Figure 5) are the representative flavanones that are found in glycoside form in nature; that form favors their intestinal absorption. They are present in citrus fruits, tomatoes and cherries [211], but unfortunately have limited solubility in water. Kanaze et al. reports that oral administration of naringenin results in a low bioavailability (5.81%) in human subjects [212]. To overcome the poor solubility and to increase naringenin use in clinical applications, several drug delivery systems have been developed. As a result, naringenin was formulated into liposomes, nanoparticles, self-nanoemulsifying drug delivery systems or nano-suspensions to assure a friendly and efficient delivery system to be used in the future [213].

It was reported that the alcoholic extracts of bergamot enriched in flavanones reduce the intestinal absorption of cholesterol and increase cholesterol excretion based on the bile acids secretion pathway [214]. Supplementation with naringenin (3%) of the high-fat diet in LDL-R-/- mice prevented hepatic steatosis by diminishing the SREBP1c expression and fatty acid synthesis, stimulated hepatic fatty acid oxidation and progression of atherosclerosis in the aortic sinus [215]. In addition, naringenin inhibited microsomal triglyceride transfer protein (MTTP) activity in HepG2 cells, reduced TG accumulation and decreased apoB100 secretion by 50–70% [216]. Naringenin (0.05%) and naringin (0.1%) reduced aortic fatty streaks in rabbits fed high-cholesterol diets by a mechanism involving reduction of hepatic ACAT activity [217]. In a clinical trial involving hypercholesterolemic patients, administration of naringin (400 mg/day, for 8 weeks) reduced plasma LDL-C and serum apoB by more than 14%, without altering plasma TG or HDL-C levels [218]. Naringenin found in bergamot inhibits LDL oxidation, initiates AMPK, modulates the activation of redox-sensitive transcription factors NF-κB induced by TNF-α and acts as ROS scavenger [219]. There are reports showing that hesperetin-induced eNOS expression increased NO production via phosphorylation of Src, Akt and AMPK in cultured EC, and consequently prevented hypertension by improving endothelial dysfunction in the hypertensive rat [220,221]. Naringenin increased the NO production diminished by the high glucose concentrations and reduced ROS production via decreased PKCβII expression in EC [187]. Certain epidemiologic studies evidence that the intake of drinks made from flavanone-rich citrus fruits improve endothelial function in humans [222].

An in vitro study demonstrated that naringenin reduces ICAM-1 in palmitic acid exposed EC by alleviating NF-κB signaling [189]. The anti-inflammatory effects of naringenin were confirmed in vivo by the same group in a model of T2D rats [189]. It was suggested that the anti-inflammatory action of flavanones from bergamot juice is due to the activation of SIRT-1 that further inhibits the transcription of pro-inflammatory cytokine IL-8 induced by LPS in EC [223,224]. Interestingly, Testai et al. observed recently that naringenin presents structural similarity with RSV, the natural SIRT-1 activator [225]. Thus, they performed an in silico study in which they detailed the binding mode of naringenin to SIRT-1. In addition, the same group demonstrated in cultured H9c2 cardiomyocytes and in vivo in 6-month-old mice that naringenin activates SIRT-1 [225].

Hesperetin attenuated VCAM-1 upregulation and adhesion of monocytes to cultured EC induced by TNF-α stimulation [220]. Administration of glucosyl hesperidin (500 mg/day) to hypertriglyceridemic patients for 24 weeks significantly reduced plasma TG and apoB levels [226]. Other human studies using capsules of hesperidin (800 mg) or naringin (500 mg) administered for 4 weeks to moderately hypercholesterolemic individuals evidenced no alterations in plasma TC, LDL-C or TG concentrations [227]. These studies reveal that the effects of flavonoids in clinical studies depend on the metabolite used, the dose, the patient population and the length of study [228].

Using LPS-stimulated PBMC from healthy subjects, Zaragoza et al. demonstrated that naringenin decreases IL-1β, TNF-α, IL-6 and IL-8 production [203]. Hsu et al. showed that naringenin extracted from *Nymphaea mexicana Zucc.* has an important inhibitory effect on MCP-1 and TNF-α production in LPS-activated RAW264.7 macrophages by decreasing the expression of iNOS and ERK phosphorylation [229]. The anti-inflammatory effects of naringenin in vivo were evaluated by Raza et al., demonstrating that naringenin treatment downregulates the NF-κB expression levels in the brains of Wistar rats after the cerebral I/R injury [230].

Although demonstrating anti-inflammatory effects, the poor water solubility and the reduced bioavailability of naringenin restricted its therapeutic use. To overcome this limitation, naringenin formulations have been developed. In a recent study, Fuior et al. encapsulated naringenin into lipid nanoemulsions (LNs), targeted to VCAM-1 exposed on the surface of activated EC [231]. They found that encapsulated naringenin decreased THP-1 monocytes adhesion and transmigration to/through activated ECs by mechanisms involving the reduction of MCP-1 and diminished nuclear translocation of NF-κB [231].

A very recent study showed that naringenin modulates the expression of miRNAs involved in fine regulation of certain oxidative and inflammatory processes. It is known that the migration inhibitory factor (MIF) has antioxidant properties and is markedly increased in Ang-II-infused mouse myocardium. Starting from these data, Liang et al. showed that miR-29b-3p and miR-29c-3p are decreased in the myocardium of Ang-II-infused MIF-KO mice, but upregulated in mouse CF with MIF overexpression or by treatment with MIF protein [232]. Interestingly, miR-29b-3p and miR-29c-3p could suppress the expression of collagen type I α1 (COL1A1), collagen type III α1 (COL3A1) and α-SMA in mouse CF by a mechanism involving the repression of the pro-fibrosis genes TGFβ2 and MMP2. Further, the authors show that naringenin could markedly reverse Ang-II-induced downregulation of miR-29b-3p and miR-29c-3p expression. Moreover, COL1A1, COL3A1, α-SMA and p-Smad3 expression were significantly decreased in Ang-II-treated CF by pretreatment with naringenin [232].

#### 2.3.4. Flavanols

**Figure 6:**
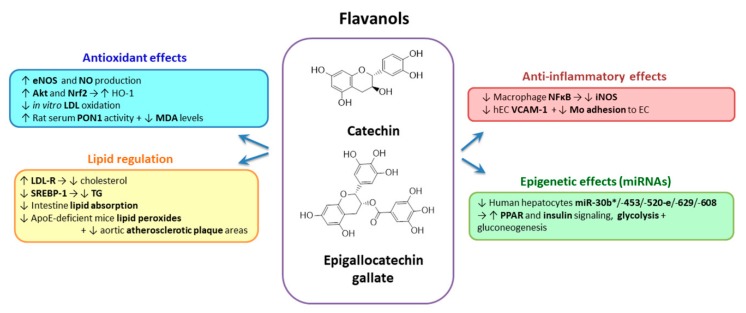
Chemical structures of catechin and epigallocatechin gallate and their molecular mechanisms of action to combat cardiovascular diseases.

*Catechins* are a family of flavonoids, subgroup flavan-3-ols (flavanol) (Figure 6). The most abundant component of catechins is epigallocatechin gallate [155]. Epigallocatechin-3-gallate (EGCG) is a catechin conjugated with gallic acid. The two or more aromatic rings of these polyphenols have at least one hydroxyl group linked by a carbon bridge which is the main source for electron donor and efficiently scavenging reactive species (singlet oxygen). Catechins are present in fresh tea leaves, red wine, broad beans, apples, pears, black grapes, apricots, strawberries, blackberries, cherries, raspberries and chocolate. Cocoa is the richest source of EGCG [155]. The poor bioavailability of catechins is a consequence of their rapid degradation under physiological conditions and their low absorption in the intestinal tract by passive diffusion. To mitigate the reduced bioavailability of EGCG and to increase its effectiveness, the consumption of 8–16 cups of green tea daily is needed, but the excessive consumption of green tea has been demonstrated to be toxic [233]. Thus, other methods to increase catechins bioavailability were developed, including encapsulation into nanostructure-based drug delivery systems, molecular modification and co-administration with some other bioactive ingredients to produce a synergistic effect [234].

Catechins present in the green tea are responsible for its cholesterol-lowering properties. It was shown that consumption of green tea in supplement formulation has cholesterol-lowering effects due to upregulation of liver LDL-R, thereby modulating the intracellular processing of lipids [235,236,237]. Their mechanisms of action involve inhibition of lipid absorption in the intestine by interfering with the micelle formation, emulsification, hydrolysis, solubilization and inhibition of squalene oxidase, a key enzyme in the hepatic cholesterol biosynthesis [238,239]. EGCG was described as the most potent inhibitor of lipid absorption in the intestine [48]. The EGCG ability to lower plasma lipids was associated with the consequential reduction of lipid peroxides levels and aortic atherosclerotic plaque areas in apoE-deficient mice [240]. Cocoa is a polyphenol-rich fruit that has been investigated for its potential to regulate the lipid metabolism. A special attention was given to the raise of HDL levels by cocoa ingestion. The results are controversial; the beneficial effects observed in vitro for cocoa epicatechins are not all confirmed by those in humans [241]. Besides the cholesterol lowering potential, catechins were described as having a TG lowering effect [242]. The mechanism responsible for decreasing TG is inhibition of hepatic lipogenesis, and more specifically, the inhibition of SREBP-1 [243].

A meta-analysis study concerning green tea consumption has indicated that it decreases the risk of CVD [244]. Many cardiovascular health benefits of flavanols have been reported, and one possible mechanism could be the modulation of homocysteine, its elevated concentrations being associated with increased CVD risk [245]. It was reported that in vitro EGCG reduces homocysteine-enhanced apoptosis by modulating mitochondrial-dependent signaling and PI3K/Akt/eNOS signaling in human EC [246]. In addition, epicatechin activates eNOS and increases NO production by inducing Ser633 and Ser1177 phosphorylation and Thr495 dephosphorylation. EGCG induces HO-1 expression in EC exposed to H_2_O_2_ through activation of Akt and Nrf2, thereby diminishing the effects of oxidative stress [247]. Many other catechins exert antioxidant properties expressed as prevention of in vitro LDL oxidation and in humans [248]. Studies on green tea found that its administration (2% in water) to diabetic rats increased serum PON1 activity [249]. These antioxidant mechanisms driven by catechins are responsible for the significant reduction of MDA levels measured in vivo and the protective action exerted on LDL and HDL. The molecular mechanisms by which EGCG or other catechins exert antioxidant effects need to be further explored in vivo.

Catechins from black tea are important scavengers of peroxyl, hydroxyl and superoxide radicals, singlet oxygen and lipid peroxides, NO and peroxynitrite radicals [250]. EGCG exerts also anti-inflammatory effects on macrophages pre-exposed to pro-inflammatory stimuli, such as LPS. EGCG blocks the disappearance of IκB from the cytosolic fraction, thereby obstructing NF-κB activation, which in turn decreases the transcription of iNOS [240]. Another in vitro study evidenced that EGCG inhibits VCAM-1 expression induced by IL-1 or TNF-α, thereby diminishing the monocytes’ adhesion to cultured human EC [250].

Catechins and their derivatives were proven to contribute to beneficial health effects by the modulation of miRNAs. In human HepG2 hepatocytes, EGCG isolated from green tea was shown to differentially inhibit the expression of a set of five miRNAs (miR-30b*, miR-453, miR-520-e, miR-629 and miR-608) that are involved in inflammatory pathways, the PPAR signaling pathway, insulin signaling, glycolysis and gluconeogenesis, oxidative phosphorylation and glutathione metabolism [251]. It was demonstrated by using 1H NMR spectroscopy, that there was direct binding of EGCG and RSV to miR-33a and miR-122 [252]. While RSV binds miR-33a and miR-122 through an A ring interaction and increases their expression levels, EGCG decreases miR-33a and miR-122 expression by direct binding through an interaction with all rings in the molecule. Wang et al. demonstrated that EGCG binds hypoxia-inducible factor 1α (HIF-1α) protein, a known transcriptional activator of miR-210, and interferes with Proline residues hydroxylation in the oxygen-dependent degradation domain [253]. While the hydroxylation of Proline residues is essential for the proteasome-mediated degradation of HIF-1α [254], EGCG binding increases HIF-1α expression and enhances miR-210 levels.

#### 2.3.5. Isoflavones

**Figure 7:**
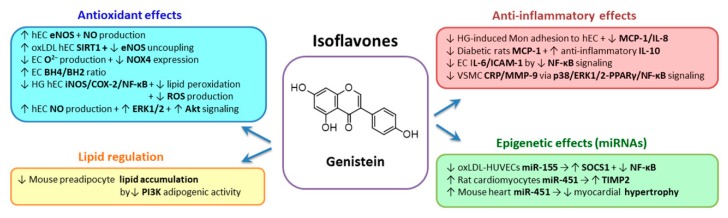
Chemical structure and protective effects of genistein in the context of cardiovascular diseases.

*Genistein* (4′,5,7-trihydroxyisoflavone) (Figure 7) is an isoflavone found in high quantities in soybeans and in many products based on soy. Genistein is also present in alfalfa and clover sprouts, barley meal, broccoli, cauliflower and sunflowers, caraway and clover seeds [255]. In humans, the plasma concentrations of genistein are dependent on the food type consumed and are highest between 2 and 12 h after ingestion of isoflavone-rich foods. Genistein has an absorption rate around 30% of the ingested dose. A small part of the intake of isoflavone aglycones (10%) is absorbed from the small intestine and metabolized in the liver. Most of the ingested isoflavone (90%) undergoes different transformations under gut microbiota action in the colon [113].

Genistein (80 mM) induced a 67% reduction of lipid accumulation in 3T3-L1 mouse preadipocytes, by inhibiting the adipogenic activity of PI3K, thereby exerting an anti-adipogenic action [113]. Opinions on the potential of isoflavones to reduce CVD risk are divided. Some epidemiologic studies highlight their protective effects on vascular EC, while other reports found no correlation between isoflavones consumption and reduction of CVD risk [256,257]. Exposure of human EC to 1–10 µM of genistein upregulated eNOS expression and enhanced NO production [258]. In addition, genistein prevented eNOS uncoupling by stimulating SIRT-1 pathway in human EC incubated with oxLDL. Furthermore, genistein diminished superoxide anion production and NOX4 expression, and improved the tetrahydrobiopterin (BH4)/dihydrobiopterin (BH2) ratio [259]. An intake of isoflavone of 50–99 mg/day was found to increase endothelial function measured as brachial flow-mediated dilation [260]. In another in vivo study, an intake of 80 mg of soy isoflavone decreased the aortic pulse-wave velocity, another marker of CVD risk [261]. Daidzein (40 µM), another member of the isoflavones group, inhibited high-glucose–induced iNOS, COX-2 and NF-κB expression in human EC, in parallel with the reduction of lipid peroxidation and ROS production [262]. Equol is a metabolite of daidzein produced by the intestinal microflora in the gut [263] and was described as activator of eNOS in EC by modulating the epidermal growth factor receptor (EGFR), the G-protein–coupled receptor GPR30 and mitochondrial ROS production [264]. Equol induced the relaxation of the rat aortic rings and stimulated endothelial NO production by activation of ERK1/2 and Akt signaling in human fetal EC [265]. In addition, soy isoflavones consumption by peri-menopausal women induced the increase of serum PON1 activity [95].

Studies from literature show that genistein exerts anti-inflammatory effects in vitro and in vivo by regulating different pro-inflammatory signaling pathways. Babu et al. showed that physiological concentrations of genistein significantly inhibit high glucose-induced adhesion of monocytes to human aortic EC and suppress MCP-1 and IL-8 endothelial production. These effects were due to genistein promoting PKA activity and were confirmed in diabetic db/db mice [266]. Genistein supplementation in diabetic rats determined a statistically significant reduction of MCP-1, while increasing the anti-inflammatory IL-10 [266]. In EC exposed to homocysteine, genistein diminished the expression of IL-6 and ICAM-1 via NF-kB inhibition [267]. In a recent study, Xu et al. evaluated the effect of genistein on inflammation induced by Ang II in vascular SMC [268]. This study demonstrates that genistein decreases CRP and MMP-9 levels in SMC by regulating p38/ERK1/2-PPARγ/NF-kB signaling pathway [268]. Besides, it was shown that genistein inhibits TNF-α secretion in LPS-activated macrophages [191].

Recent studies show that genistein modulates the expression of miRNAs involved in inflammatory processes. It was reported that genistein pretreatment of HUVECs reduces in a dose-dependent manner the oxLDL-induced expressions of E-selectin, P-selectin, MCP-1, IL-8, VCAM-1 and ICAM-1. Further analyses established that the mechanism of action consists of genistein inducing reduction of miR-155 levels and elevation of the suppressor of cytokine signaling 1 (SOCS1) expression that further induces the inhibition of NF-ĸB signaling pathway in HUVECs [269]. A recent study of Gan et al. showed that genistein can inhibit isoproterenol-induced cardiac hypertrophy by increasing miR-451 and the tissue inhibitor of metalloproteinases 2 (TIMP2) expression (a miR-451 target gene), both in vitro in H9C2 embryonic rat cardiomyocytes and in vivo in isoproterenol-induced myocardial hypertrophy in mice [270].

#### 2.3.6. Anthocyanidins

**Figure 8:**
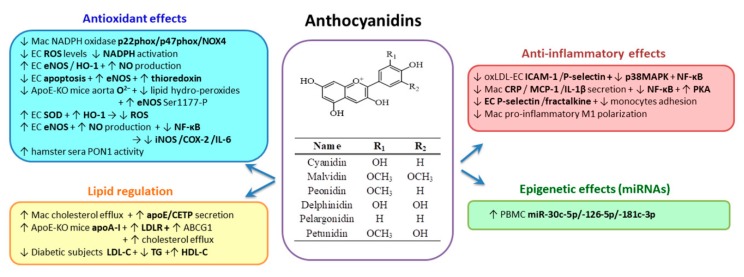
Anthocyanidins’ classifications, chemical structures and beneficial effects for improving cardiovascular disease outcomes.

*Anthocyanins* and *anthocyanidins* are glycosylated, poly-hydroxy or poly-methoxy derivatives of flavylium cations (2-phenylchromenylium) (Figure 8). In nature, about 702 different anthocyanins and 27 anthocyanidins are present. They are water-soluble plant pigments that give red, purple or blue coloration to many fruits, flowers and leaves. The widely distributed anthocyanidins in human foods are: cyanidin, delphinidin, pelargonidin, peonidin, malvidin and petunidin. They are found in many berry fruits, eggplantss, red onion, purple cabbage and black rice [271]. Anthocyanins’ bioavailability has quite large inter-individual variability, and is influenced by the food processing, availability of the enzymes involved in anthocyanins metabolism and the composition of the gut microbiota that metabolize anthocyanins [272]. After ingestion, anthocyanins appear rapidly in the circulation, reach the maximal concentration of ~100 nM within 1.5h and disappear from the bloodstream by 6h post consumption. The bioavailability of anthocyanins is very low (1%), but it is increased by the absorption of their active metabolites (12.4%) that were recently identified [271].

Anthocyanines/anthocyanidines and their metabolites ameliorate endothelial dysfunction and diminish the CVD risk [273]. Anthocyanins and flavonoids from bilberry (*Vaccinium myrtillus*) alcoholic extract can reduce the lipid deposits from the artery wall by inducing the cholesterol efflux from lipid-loaded macrophages through a mechanism involving increased secretion of apoE and CETP [274]. Anthocyanins from black elderberry (*Sambucus nigra*) extract induce expression of apoA-I and LDL-R in apoE-deficient mice [275]. Administration of 320 mg/day anthocyanins to diabetic patients for 24 weeks improved their lipid profiles by significantly decreasing LDL-C and TG, and increasing HDL-C [276].

In parallel with the lipid-lowering effects, anthocyanins show antioxidant properties. Thus, the alcoholic extract of bilberries reduces the expression of NADPH oxidase subunits (p22phox, p47phox and NOX4) in lipid-loaded macrophages derived from THP-1 monocytes [274]. Mulberry (*Morus alba*) leaves have considerable amounts of flavonoids and anthocyanins. Treatment of human EC, pre-exposed to pro-inflammatory stimuli, with *Morus Alba* extract, inhibited the intracellular ROS levels due to reduction of NADPH oxidase activation [64]. Cyanidin-3-O-b-glucoside (C3G), a metabolite of cyanidin, has been shown to upregulate eNOS and HO-1 expression in a dose-dependent manner in EC, in parallel with an increase of NO production. The mechanism involves eNOS phosphorylation at Ser1179 and dephosphorylation of Ser116 [277,278]. Thioredoxin is one of the key regulators of intracellular redox status and it protects EC against oxidative stress. It was reported that nonaglycone cyanidin reduces TNFα–induced apoptosis and upregulates eNOS and thioredoxin in EC [278]. Other pathways involved in EC protection by cyanidins are Akt, ERK1/2 and Src kinase positive regulation. In vivo studies show that C3G intake (2 g/kg diet for 8 weeks) diminished the area of atherosclerotic plaques and alleviated the endothelium-dependent relaxation in fat-fed apoE-deficient mice. The underlying mechanisms of these benefic effects are: the decrease of superoxide and lipid hydroperoxide generation; the increase of Ser1177 phosphorylation in eNOS protein from the aorta; and the increase of ABCG1 expression that facilitates cholesterol efflux [279,280]. It was demonstrated that malvidin and its metabolites reduce ROS levels by upregulating SOD and HO-1 in EC [281]. In parallel, they induce eNOS expression, increase NO production and reduce peroxynitrite-induced NF-kB activation that further decreases the levels of pro-inflammatory mediators such as iNOS, COX-2 and IL-6 [282]. Anthocyanins combined with gallocatechins from *Hibiscus sabdariffa* extract, increase PON1 activity in hamster sera in a dose-response manner [283]. Alcoholic extract of maqui berry, another black fruit, decreases the levels of lipid peroxides and F2-isoprostanes in humans [284]. A study conducted in women shows that the dietary intake of anthocyanins is accompanied by a lower carotid-femoral pulse wave velocity and carotid intima-media thickness [285].

Chen et al. demonstrated that pretreatment with delphidin determines the reduction of ICAM-1 and P-selectin expression in oxLDL-activated EC, resulting in an inhibition of monocytes adhesion and transmigration [286]. These anti-inflammatory effects were determined by the inhibition of oxidative stress, mitigation of p38 MAPK expression and inhibition of NF-κB [286]. The bilberry extract diminishes the secretion of CRP, MCP-1 and IL-1β in lipid–loaded macrophages. These effects are driven by the inhibition of NF-κB and activation of PKA signaling pathways [274]. The *Morus alba* extract downregulates the pro-inflammatory molecules P-selectin and fractalkine, decreasing monocytes adhesion to EC [64]. In a recent study, Lee et al. demonstrated that anthocyanin-rich blackcurrant extract exerts anti-inflammatory action by repressing the pro-inflammatory M1 polarization of mouse bone marrow-derived macrophages and human THP-1 cells [287].

The effects of anthocyanins and their metabolites on miRNAs expression are still largely unknown, only a few studies having been published. Rodriguez-Mateos et al. performed, recently, a nutrigenomic study to explore the mechanism of action of anthocyanins in vivo [288]. They analyzed mRNAs and miRNAs in PBMC isolated from healthy volunteers at the beginning and the end of a 28-day period of anthocyanins-enriched vitamin mix or blueberry consumption. The results of the microarray analysis in PBMC showed that a daily blueberry consumption led to differential expression (>1.2-fold) of 608 genes and three miRNAs (miR-30c-5p, miR-126-5p and miR-181c-3p). The most striking finding was a 13-fold increase of miR-181c expression evidenced in PMBC. Specific patterns of 13 metabolites were proven as independent predictors of mRNA expression alteration, and pathway enrichment analysis revealed significantly modulated biological processes involved in cell adhesion, migration, immune response and cell differentiation [288].

Anthocyanins are well tolerated and have no side effects, but their bioavailability is rather low, and they are rapidly transformed into phenolic acid derivates [289]. For this reason, additional experiments should be performed to develop new formulation of these natural active compounds to increase their protection, bioavailability and efficiency in vivo. Further studies are also desirable to assess the clinical efficiency of anthocyanins in different populations and to evaluate their benefic effects exerted on arteries affected by atherosclerosis.

### 2.4. Guaiacols Group

**Figure 9:**
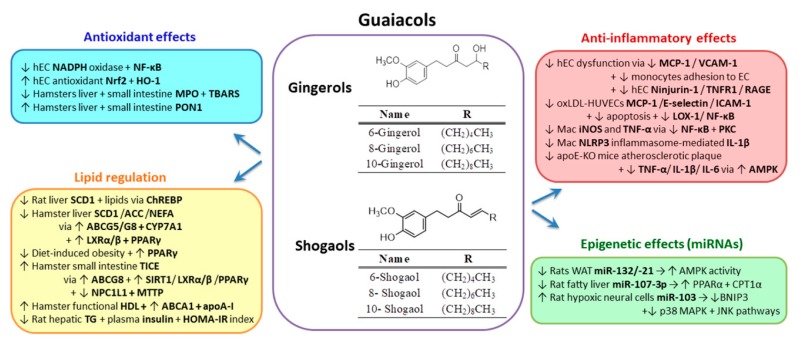
Guaiacols’ classifications, chemical structures and molecular mechanisms of action to reduce cardiovascular diseases.

*Gingerols* and *shogaols* are the most abundant active compounds of ginger (*Zingiber officinale*) rhizomes that are used since old times in the treatment of various symptoms and as dietary supplement in drinks and food products [290,291]. Gingerols differ in the length of their unbranched alkyl side chains, [6]-gingerol being the most abundant type in fresh ginger root, followed by [10]-gingerol and [8]-gingerol (Figure 9). Dehydration of these major gingerols generates the corresponding shogaols (Shao et al., 2010). The low solubility of the orally ingested gingerols generates their low bioavailability. These compounds are not completely free of side effects due to their interactions with other pharmaceutical active compounds, the gingerols acting as bioenhancers of certain drugs. Possible technological solutions for enhancing gingerols solubility and bioavailability, and preventing their harmful interactions comprise the microemulsions and nanocarriers/nanoparticles formulations of gingerols. Some liposomal ginger products were developed to increase their bioavailability. These structures are not degraded in the stomach, can enter liver cells, but their benefic effects remain to be demonstrated by in vivo studies [292].

Ginger extract (GEx) exerts lipid–lowering effects, reduces stearoyl CoA desaturase 1 (SCD1) gene expression and accumulation of lipids in the liver of rats fed a fructose diet through a pathway mediated by hepatic carbohydrate response element-binding protein [293]. Recently, it was shown that a GEx, with very well characterized composition in gingerols and shogaols, diminishes the fatty acid production in hyperlipidemic conditions by reducing SCD1, ACC and non-esterified fatty acids levels in the liver and plasma of hyperlipidemic hamster [294]. The lipid-lowering properties of gingerols and shogaols can be also explained by the enhancement of hepatic cholesterol excretion into bile fluids, based on the induction of ABCG5/G8 and CYP7A1 expression in the liver of hyperlipidemic hamster. Lei et al. reported that the treatment with gingerol and shogaol-enriched GEx upregulates hepatic CYP7A1 [295]. The increase of ABCG5/G8 and CYP7A1 levels can be due to the induction of their transcription regulators LXRα/β and PPARγ which are stimulated by the decrease of endoplasmic reticulum stress [294]. The upregulation of PPARγ by GEx was also detected by Misawa et al., who showed that GEx attenuates diet-induced obesity and improves exercise endurance capacity by activation of the PPARγ pathway [296]. Gingerols and shogaols stimulate TICE through stimulation of ABCG8 gene and protein expression in the small intestine, due to the upregulation of SIRT1-LXRα/β-PPARγ pathway [117,295]. In addition, the treatment with gingerol and shogaol-enriched GEx induces the downregulation of Niemann-Pick C1-Like 1 (NPC1L1) and MTTP in the small intestine of hamster [295]. In parallel, GEx stimulates the small intestine to produce functional HDL by restoring ABCA1 levels and apoA-I quality and quantity through inhibition of the oxidative stress. It was reported that these processes happening in the small intestine and liver are associated with the reduction of the aortic valves lipid-deposits [117].

GEx active constituents have proven to be effective in exerting antioxidant effects on dysfunctional EC in culture by decreasing the expression of NADPH oxidase subunits, inhibiting NF-κB, activating the antioxidant Nrf2 and HO-1. GEx acts in vivo as antioxidant under dyslipidemic conditions by reducing MPO and thiobarbituric acid reactive substances (TBARS) levels and increasing PON1 levels in the liver, small intestine and plasma of hyperlipidemic hamsters [117,294].

Ginger is known since ancient times for its anti-inflammatory effects that ameliorate various diseases [297]. Recent data evidence the GEx potential and its major components, 6-gingerol and shogaol, to exert anti-inflammatory effects in EC by reversing TNFα-induced EC dysfunction. Their mechanisms of action involve reduction of MCP-1, VCAM-1 and monocytes adhesion to EC, due to the decrease of Ninjurin-1, TNFα receptor 1 and RAGE expression [298]. Wang et al. demonstrated that 6-shogaol decreases MCP-1, E-selectin and ICAM-1 and the apoptosis of HUVECs exposed to oxLDL by inhibiting LOX-1, oxidative stress and NF-κB signaling [299]. Beneficial effects of 6-gingerol and 6-shogaol were demonstrated also in macrophages. In LPS-stimulated macrophages, 6-gingerol decreased iNOS and TNF-α expression by inhibiting NF-κB and PKC signaling [300]. In another study, 6-shogaol significantly inhibited the canonical NLRP3 inflammasome-mediated IL-1β secretion in THP-1 macrophages stimulated with LPS [301]. In a recent in vivo study, Wang et al. demonstrated that administration of 6-gingerol to fat-fed apoE-deficient mice determines the reduction of atherosclerosis, expressed as decreased plaque formation and reduced levels of pro-inflammatory cytokines (TNF-α, IL-1β, and IL-6) by a mechanism mediated in part by AMPK activation [302].

Knowing the GEx pleiotropic effects, several groups investigated its potential to modulate epigenetic factors associated with ncRNA. As the main active ingredient of ginger, 6-gingerol, was shown to significantly improve lipid metabolism abnormalities in adult rodents. A few studies have reported its molecular effects on age-related non-alcoholic fatty liver disease (NAFLD), as well as on epigenetic factors. In an in vivo recent study in aged rats, Li et al. demonstrated that 6-gingerol brought to normal the hepatic TG content, plasma insulin and HOMA-IR index of ageing rats [303]. Mechanistically, they showed that 6-gingerol modulates lipid metabolism by increasing β-oxidation and decreasing lipogenesis, through activation of liver PPARα and carnitine palmitoyl-transferase 1α (CPT1α), and inhibition of diacylglycerol O-acyltransferase 2 (DGAT-2) expression at translational level, but not at transcriptional level, to ameliorate ageing-associated hepatosteatosis. The authors further analyzed miRNAs targeting PPARα and CPT1α genes and reported that ageing significantly increased hepatic miR-34a expression in rats. Interestingly, 6-gingerol showed minimal effect on hepatic miR-34a. Additionally, 6-gingerol significantly decreased hepatic miR-107-3p level, thereby increasing PPARα and CPT1α genes [303]. Kim et al. investigated molecular factors involved in lipid metabolism and inflammation of the white adipose tissue (WAT), including miRNAs, modulated by GEx in Sprague–Dawley rats fed a high-fat diet [304]. They reported that GEx reduced body weight and WAT mass, mRNA levels of adipogenic genes, PPARγ, adipocyte protein 2 (aP2), as well as pro-inflammatory cytokines (TNFα, IL-6 and MCP-1). Obese rats expressed increased expression of miR-21 and miR-132 in WAT. Additionally, this report showed downregulated expression of miR-132 and miR-21 in WAT of rats after GEx administration, in parallel with a greater AMPK activity. The authors assumed that the reduced miR-21 and miR-132 levels could be associated with the post-translational regulation of genes involved in adipogenesis and inflammation in high-fat diet fed rats [304].

It has been reported that some phenolic compounds such as curcumin, RSV, quercetin, EGCG and genistein can interfere with assays via a number of different mechanisms (are potential pan-assay interference compounds-PAINS) and/or are considered invalid metabolic panaceas (IMPs) [305,306]. We recommend that this “warning bell” should be very seriously taken in consideration by the researchers that intend to extend the research in the field of phytochemicals therapeutic potential. The present review has taken in consideration only the phenolic compounds with known molecular mechanisms in vitro and in vivo, precisely to support their specific actions, and does not propose them as panacea.

## 3. Conclusions

This review amassed consistent knowledge concerning the mechanisms of action of the phenolic compounds, which ascertain their therapeutic potential to prevent or treat CVD. Phenolic compounds can regulate lipid metabolism and balance the oxidative and inflammatory stress through various epigenetic, transcriptional and translational mechanisms that have been demonstrated in vitro and in animal models; some of them have been confirmed in humans.

Phenolic compounds have various pharmacological properties and the present review highlighted those having robust potential to amend dyslipidemia or to diminish the oxidative and inflammatory stress, important risk factors for CVD. Thus, remarkable lipid-regulatory properties have the phenols from hydroxycinnamic acid, flavanols, anthocyanidines and guaiacols groups. They inhibit the lipid absorption in the small intestine; stimulate the cholesterol efflux from atheroma and cholesterol excretion through gallbladder or small intestine; and imped de novo lipid synthesis in the liver. The molecular mechanisms responsible for these effects involve activation of transcription regulators (SIRT-1, LXRs, PPARs). Important antioxidant and anti-inflammatory effects are described for almost all presented phenolic compounds, the main mechanisms of action being inhibition of NLRP3 inflammasome and NF-κB; activation of Nrf2 and Akt, and the consequent stimulation of eNOS and antioxidant enzymes, and inhibition of NADPH oxidase. All these processes are susceptible to be regulated at the epigenetic level, an increasing number of miRNAs modulating them being depicted. Interestingly, certain phenolic compounds, such as resveratrol and naringenin, bind directly to the genes to regulate various proteins synthesis.

Most of these findings come from experiments performed either in vitro or in vivo (experimental animals). Many of these outcomes have been consistently achieved from clinical studies, but some of them still need to be proved in humans. More studies are needed to widen the knowledge concerning the molecular actions of the phenolic compounds; some of them are not completely understood or are still controversial. Since the results obtained until now on improving the human health are promising, more studies in humans are needed to validate the beneficial effects of phenolic compounds in CVD. Additional clinical trials are warranted, but with improvement and standardization of the study design, formulations and doses to be assessed. Further investigations are necessary using larger and longer studies to better define the therapeutic role of phenolic compounds.

Herbal medication has lower adverse effects compared to synthetic drugs and thus presents the advantage that they can be used for a longer period of time. Many of the plant phenolic compounds exert their effects through different mechanisms, and we might expect additive effects when taken in combination, but this has to be scientifically proven. In many cases, it was demonstrated that the biologically active compounds are more efficient in plant extracts compared to the purified molecular components, the data supporting the synergistic action of plants’ active compounds. Analyses comparing intake of phenolic compounds in different formulation (powder capsules, alcoholic extracts or encapsulation in nanocarriers) have produced conflicting results. Thus, greater attention should be given to combinations of phenolic compounds, their doses and formulations for administration. Further studies are needed to develop new forms of delivery for such natural bioactive compounds to improve compliance of patients at risk for CVD, and ensure their increased bioavailability, thereby creating more efficient products than the commercially available ones at present.

In the present review we have focused on the molecular mechanisms of action of the phenolic compounds that target dyslipidemia, oxidative and inflammatory stress, the main risk factors in the atherosclerotic process. We hope that we convinced the reader that their multiple benefic effects warrant their use as CVD remedies, complementarily to allopathic drugs. Consumption of foods containing natural phenolic compounds should be encouraged for people with low to moderate CVD risks; those who are preventive, and those who are at the same time on the prescription drugs to lower the incidence of fatal cardiovascular events.
